# The Interplay of Spontaneous Pupil‐Size Fluctuations and EEG Power in Near‐Threshold Detection

**DOI:** 10.1111/psyp.70035

**Published:** 2025-03-16

**Authors:** Veera Ruuskanen, C. Nico Boehler, Sebastiaan Mathôt

**Affiliations:** ^1^ Department of Experimental Psychology University of Groningen Groningen the Netherlands; ^2^ Department of Experimental Psychology Ghent University Gent Belgium

**Keywords:** arousal, EEG, near‐threshold detection, pupil size, visual perception

## Abstract

Detection of near‐threshold stimuli depends on the properties of the stimulus and the state of the observer. In visual detection tasks, improved accuracy is associated with larger prestimulus pupil size. However, it is still unclear whether this association is due to optical effects (more light entering the eye), correlations with arousal, correlations with cortical excitability (as reflected in alpha power), or a mix of these. To better understand this, we investigated the relative contributions of pupil size and power in the alpha, beta, and theta frequency bands on near‐threshold detection. We found that larger prestimulus pupil size is associated with improved accuracy and more stimulus‐present responses, and these effects were not mediated by spectral power in the EEG. Pupil size was also positively correlated with power in the beta and alpha bands. Taken together, our results show an independent effect of pupil size on detection performance that is not driven by cortical excitability but may be driven by optical effects, physiological arousal, or a mix of both.

## Impact Statement

Our research offers a unique perspective on near‐threshold visual detection by focusing on the often overlooked effect of pupil size. We show that the pupil has an independent effect on visual sensitivity that is not mediated by cortical excitability, as measured by power variations in different EEG frequency bands. Our results highlight pupil size changes as an important yet poorly understood factor in visual processing.

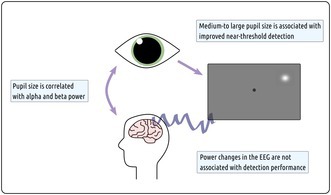

Whether a stimulus is perceived or not depends on the external properties of the stimulus and the internal state of the observer. Internal factors are especially relevant at the threshold of detection, where the same stimulus is sometimes perceived and sometimes not. In this context, increased detection rates are associated with larger pupils (Mathôt and Ivanov 2019) and changes in the frequency spectrum of the electroencephalogram (EEG), especially in prestimulus alpha power (Ergenoglu et al. 2004). These effects are related but do not necessarily reflect the same underlying mechanism. Increased detection rates can result from optical effects (pupil dilation increases visual sensitivity by allowing more light into the eye) as well as neural effects (enhancement of cortical excitability increases visual sensitivity by changing how visual input is processed). Here, we attempt to dissociate the two.

The state of the brain affects sensory perception. One aspect of ‘brain‐state’ is cortical excitability, indexed by oscillations in the EEG, especially the alpha frequency band (8–13 Hz). Lower power in the alpha band (alpha suppression) has been suggested to indicate higher excitability (Lange et al. 2013; Romei et al. 2008). Changes in both prestimulus alpha power (measured at occipital electrodes) (Benwell et al. 2017; Benwell et al. 2022; Ergenoglu et al. 2004; Limbach and Corballis 2016; Melcón et al. 2024; Samaha et al. 2017) and alpha phase (Busch et al. 2009; Mathewson et al. 2009; van Dijk et al. 2008) have been associated with changes in near‐threshold detection performance. Specifically regarding alpha *power*, lower prestimulus levels are linked to an increased hit rate (Ergenoglu et al. 2004; van Dijk et al. 2008). In terms of signal detection theory (SDT), alpha‐power suppression seems to lead to increased hit rates by modulating the criterion (i.e., the decision threshold) rather than changing the observer's sensitivity (d') (reviewed in Iemi et al. 2017; Samaha et al. 2020). But regardless of the exact mechanism, there is clear evidence that neural oscillations related to cortical excitability are linked to detection performance.

Another—largely neglected—factor that influences detection performance is pupil size. Studies manipulating pupil size through changes in background brightness show improved detection (i.e., increased accuracy as well as hit rate) of faint stimuli in the visual periphery when pupils are larger (Eberhardt et al. 2022; Mathôt and Ivanov 2019). Similarly (although to a lesser extent) smaller pupils are related to improved discrimination performance (Campbell 1957; Campbell and Gregory 1960; Mathôt and Ivanov 2019; Woodhouse 1975). Larger pupils benefit detection by increasing the amount of light that enters the retina, leading to a better signal‐to‐noise ratio. On the other hand, smaller pupils benefit discrimination by focusing incoming light on the fovea, where visual acuity is highest (Mathôt 2020). Interestingly, in an experiment involving both detection and discrimination, average pupil size was found to be larger in the detection condition, despite no other obvious differences (i.e., in difficulty or background brightness) between conditions (Mathôt and Ivanov 2019). This suggests that the pupil is able to flexibly adjust to a size that is optimal to the demands of the task at hand (for further discussion see Vilotijević and Mathôt 2023).

The pupil does not only respond to changes in visual input but also to internal factors such as cognitive activity. Pupil‐size changes have been observed in a variety of tasks and contexts, such as problem solving, working memory, decision making, and attention (Alnæs et al. 2014; Hess and Polt 1960; Hess and Polt 1964; Kahneman and Beatty 1966; Keene et al. 2022). In general, increases in task difficulty or processing load lead to pupil dilation irrespective of the specific cognitive processes involved, and larger pupils are often associated with better performance (for reviews see Beatty 1982; Laeng et al. 2012; Loewenfeld 1958; Mathôt 2018; Vilotijević and Mathôt 2023). These types of pupil responses are suggested to be mainly driven by changes in arousal (Bradley et al. 2008; Gilzenrat et al. 2010; Grujic et al. 2024; Unsworth and Robison 2018). This follows from the link between pupil size and activity in brain regions associated with arousal regulation, most notably the locus coeruleus (LC) (Joshi et al. 2016; Joshi and Gold 2020; Murphy et al. 2014). In addition to arousal regulation via norepinephrine release, the LC is implicated in other high‐level cognitive functions, such as attention and behavioral control (Aston‐Jones et al. 1999; Aston‐Jones and Cohen 2005). Due to the link between pupil size and LC activity, pupil size is often used as a marker of arousal.

Furthermore, fluctuations in pupil size are correlated with alpha power both during task performance and at rest. For instance, in an auditory oddball task, increased alpha prior to stimulus presentation is associated with decreased stimulus‐evoked pupil dilation (Hong et al. 2014). Similarly, both in visual detection tasks (Pilipenko and Samaha 2024) and perceptual decision‐making tasks (Podvalny et al. 2021), alpha power and pupil size show a positive correlation within the prestimulus interval. Conversely, in an auditory discrimination task (Waschke et al. 2019) a negative correlation was observed, whereby larger pupil size was associated with decreased alpha activity. During rest, or inactive wakefulness, studies show associations between alpha power and various aspects of pupil dynamics. Montefusco‐Siegmund et al. (2022) have linked alpha power with high‐frequency pupil diameter fluctuations and the rate of change in pupil size, while Ceh et al. (2020) found correlations with both the average and variance of pupil diameter. Specifically, they report a positive relationship between alpha power and average pupil diameter, contrasted by a negative relationship with the variance in pupil diameter. Others show both negative (Dahl et al. 2022) and more complex patterns of relationships dependent on time‐course and cortical region (Pfeffer et al. 2022). Relevant for the current study is that in the occipital areas, the full alpha and beta (8–32 Hz) frequency range was found positively correlated with pupil size, highlighting the nuanced relationship between pupil size and alpha power. Overall, despite considerable variability between studies, the general trend indicates a positive correlation between pupil size and alpha power, especially during visual detection tasks.

Taken together, previous research has shown that both alpha power and pupil size are associated with detection performance but in different ways: alpha power and accuracy in detection tasks show a negative correlation, while pupil size and accuracy show a positive correlation. Furthermore, as outlined above, alpha power and pupil size are positively correlated (in visual detection tasks, with the relationship being more variable in other contexts). This pattern of results—a triangle of seemingly inconsistent correlations—suggests that alpha power and pupil size affect detection performance through different routes. Indeed, in a study on visual detection and confidence across multiple stimulus contrast levels, alpha power and pupil size were shown to have dissociable behavioral effects, whereby alpha modulated detection criterion (i.e., the likelihood to report the presence of a target regardless of whether a target was actually present) while pupil size modulated detection sensitivity (Pilipenko and Samaha 2024). More specifically, lower prestimulus alpha power was associated with a lower criterion across all contrast levels, and larger prestimulus pupil size was associated with improved sensitivity, especially at higher contrast levels. However, in another study on perceptual decision‐making where pupil size and MEG were simultaneously recorded, a slightly different pattern emerged. Pupil size was positively correlated with hit rate and showed an inverted‐U shaped relationship with sensitivity, while alpha power was associated with both an improved hit rate and higher sensitivity (Podvalny et al. 2021).

In the two experiments described above, as in many other studies on pupil‐linked effects, pupil size is assumed to function simply as a marker of arousal. Following this assumption, the large‐pupil advantage observed in near‐threshold detection would be attributed to arousal variation. However, this account overlooks the potential optical effect of pupil size itself. As discussed above, pupil size controls how light falls on the retina, leading to changes in visual acuity, which are likely to play a role in determining detection performance.

Furthermore, both spontaneous and experimentally induced changes in pupil size have been shown to affect neural signatures of visual processing. These include the amplitude of the C1 component, associated with processing in the primary visual cortex (Bombeke et al. 2016), activity patterns in the beta frequency range (Mathôt et al. 2023), and the strength of steady‐state visual evoked potentials (SSVEPs) (Suzuki et al. 2019). These findings suggest that pupil size changes optimize vision at the earliest stages of visual processing. Still, research explicitly investigating pupil‐related effects on perception is relatively scarce.

Moreover, the exact meaning and nature of the relationship between pupil size and arousal beyond the LC–NE system remain undefined (Grujic et al. 2024). Similarly, different studies may define arousal and its neural markers differently, making it difficult to draw firm conclusions on how arousal and its constituents affect behavior (see Discussion for more details). Here, we use the term arousal to refer to a general level of alertness, which we believe to have both a physiological and neuromodulatory effect, and as such, may be reflected in EEG (in addition to pupil size). However, for the sake of completeness and consistent with the terminology used in key studies on alpha‐power suppression (Iemi et al. 2017; Pilipenko and Samaha, 2024; Samaha et al. 2020), we also discuss EEG power effects as reflections of changes in cortical excitability.

In the current study, we aim to further investigate the relative contributions of pupil size and neural activity on near‐threshold detection, with a focus on the often‐overlooked effect of the pupil. In terms of neural activity, in addition to alpha power, we will also analyse activity patterns in the beta and theta frequency bands. We expect to find that both larger prestimulus pupil size and lower prestimulus alpha power are associated with better detection performance (i.e., improved accuracy). To investigate the relative contributions of pupil size and power in the different frequency bands, we will conduct a mediation analysis. However, due to the exploratory nature of this study, we do not formulate specific hypotheses on the nature or direction of possible mediation effects.

## 1 Methods

### 1.1 Open‐Practices Statement

Experimental materials, raw data, and analysis scripts are available on the open‐science framework (https://osf.io/9p563/). The study was not preregistered.

### 1.2 Participants

Nineteen participants with normal or corrected‐to‐normal vision participated in the experiment. The sample size was not predetermined and is arguably moderate compared to other studies, but reflects the number of participants that VR was able to test during a research visit to the lab of NB. The two participants who did not have normal vision wore soft contact lenses (not glasses) during the recording. After preprocessing, three participants were excluded from the analysis due to poor data quality (10% or more of epochs marked bad and high amounts of noise identified already during recording). Data exclusion was conducted based on visual inspection, and it was decided before any further analyses had been conducted. The mean age in the final sample (*N* = 16) was 24 years, and there were 5 males and 11 females.

All participants provided written informed consent prior to participation. The experiment was approved by the ethics committee of the psychology department at the University of Gent (study code: SEP 2021–215). Participants received a €25 monetary compensation for participating in the full experiment (€15/h during task performance and €10/h during EEG preparation).

### 1.3 Detection Task

Participants completed a near‐threshold visual detection task consisting of reporting the presence of a faint peripherally presented stimulus. The experiment and stimuli were created with and controlled by OpenSesame (version 3.3.9, Lentiform Loewenfeld) (Mathôt et al. 2012).

**FIGURE 1:**
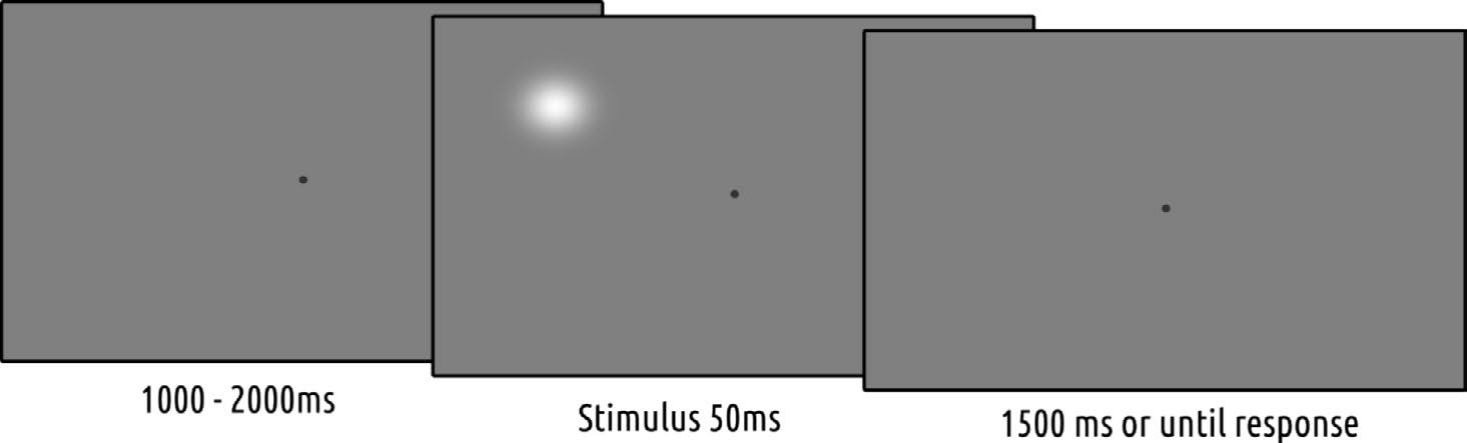
Detection task. The stimulus was present on 50% of the trials. This illustration is not to scale. Stimulus contrast, as shown, is higher than in the actual experiment for illustration purposes.

All stimuli were presented on a gray (RGB = 128, 128, 128) background with a luminance of 18.5 cd/^2^m^2^. A circular gray fixation dot (RGB = 89, 89, 89) with a size of 0.3° of visual angle (20 px) was maintained in the center of the screen throughout the experiment (Figure 1).

The to‐be‐detected target stimulus was a white luminance patch (RGB = 255, 255, 255) with a Gaussian envelope with a standard deviation of 0.46° (30 px) (Figure 1). The luminance of the stimulus at full contrast was 36.4 cd/^2^m^2^. However, the contrast of the stimulus was adjusted with a staircase procedure to keep overall accuracy fixed at 75%. Consequently, stimulus luminance during the task was lower. Possible values ranged between 5% and 25% for contrast. Stimulus location was determined by drawing a random angle between 0° and 360° with a fixed eccentricity of 9.11° of visual angle (500 px).

Each trial started with a variable target‐onset time drawn from a flat distribution between 1 and 2 s. Next, on half of the trials, the target stimulus was flashed for 50 ms. After target onset, each trial lasted for an additional 1.5 s. Therefore, total trial length varied between 2.5 and 3.5 s. Since the fixation dot was maintained on the screen throughout the experiment, it was not clear to the participants when an individual trial started or ended. Participants were instructed to press the spacebar on the keyboard whenever they detected the target. Upon keypress, the fixation dot changed to a dot with a Gaussian envelope for 10 ms to indicate to the participant that a response was recorded.

The task consisted of a practice phase and an experimental phase. There were 12 trials in the practice phase and 1040 trials in the experimental phase. The trials of the experimental phase were divided into 16 blocks, separated by self‐paced breaks. During 4 of the blocks (block numbers 1, 5, 9, and 13), the staircase procedure was applied to vary the properties of the target stimulus as described above to keep overall accuracy fixed at 75%. These blocks consisted of 50 trials each and were not included in the analysis. The remaining 12 blocks consisted of 70 trials each, yielding a total of 840 experimental trials that were included in the analysis. Participants received feedback on their average accuracy at the end of each block. One block lasted approximately 3 to 4 min.

The experiment was presented on a 27‐in. (68.58 cm) Benq LED monitor (model number: XL2411Z) running at a refresh rate of 100 Hz and a 1920 × 1080 resolution. The viewing distance was approximately 100 cm for all participants.

### 1.4 EEG Acquisition

The EEG signal was acquired from 64 Ag/AgCI scalp electrodes, positioned according to the international 10–20 system, as well as three external electrodes, placed on the mastoids and under the left eye. The signal was recorded at 512 Hz, using a BioSemi ActiveTwo amplifier with its built‐in CMS‐DRL recording logic, and the associated software (BioSemi B.V.). No online referencing or filtering was done (other than the standard anti‐aliasing filter related to the sampling rate). EEG was measured continuously from the beginning to the end of the experimental task.

### 1.5 Pupil Size Measurement

Pupil size was measured with an Eyelink 1000 eye tracker (SR Research Ltd., 2022) with a 35 mm lens. An initial 9‐point calibration procedure was performed at the beginning of the experiment, and a recalibration was performed halfway through. A drift correction was performed at the beginning of each block. Pupil size was measured from the beginning to the end of each trial. Participants rested their head on a chinrest throughout the experiment, keeping the distance to the eye tracker fixed at 60 cm.

### 1.6 Procedure

Upon arrival in the lab, participants received information about the study in both verbal and written form, after which they signed the informed consent form. After the informed consent procedure, the EEG cap was placed on the participants' head, filled with gel, and optimized until all electrode offsets were low (< 10 kΩ) and stable, and the eye tracker was calibrated.

Next, participants completed the detection task. Specific task instructions were given verbally before the informed consent procedure and again on the screen at the beginning of the task. Participants were tested in a Faraday cage with constant, dim illumination (13 lx). The light source in the room was a small table lamp behind the screen, which fully covered it from direct view, yielding a diffuse illumination.

The whole experimental session, including informed consent, preparation, task performance, and debriefing, took approximately 2 h per participant.

### 1.7 Preprocessing

#### 1.7.1 EEG

EEG preprocessing was conducted in the MATLAB (version 9.11 R2021b) (MATLAB 2021) toolbox EEGLAB (version 14.1.1) (Delorme and Makeig 2004). The data was visually inspected for exceptionally large artifacts (such as those resulting from movements of the cap), which were removed if found. Then, the data was rereferenced to the mastoids, and a 0.1 Hz high‐pass filter was applied. One subject (subject 6) had periods of missing signal in the mastoid channels, likely due to the electrodes not being properly attached, so for this participant an average reference was used instead. Independent component analysis (ICA) was run for each participant, and topographic maps of the components were created. The signal was reconstructed without blink‐related components (identified through visual inspection from the ICA topographic maps; one component was found for each participant), and the components were then rejected following a visual inspection of the reconstructed signal. Possible bad channels (noted during recording) were excluded from the ICA. They were subsequently interpolated and reincorporated into the data. One channel was interpolated for three participants (P2: subjects 1, 3, and 6) and two channels were interpolated for one participant (FC2 and CP2: subject 7). The data of all other participants did not require channel interpolation.

Next, data was exported from MATLAB and imported into eeg_eyetracking_parser, a Python library for the analysis of combined EEG and eye tracking (Mathôt et al. 2023), which in turn relies on MNE (Gramfort et al. 2013), Autoreject (Jas et al. 2017), EyeLinkParser (Mathôt and Vilotijević 2022), and DataMatrix. Data was downsampled to 100 Hz. When extracting epochs, the Autoreject algorithm (Jas et al. 2017) was applied to detect and interpolate bad epochs and channels that may not have been identified in the previous preprocessing steps.

Autoreject relies on cross‐validation to estimate the optimal peak‐to‐peak threshold in the data (for details, see Jas et al. 2017). Finally, trials where eye position deviated more than 3° of visual angle from the center for over 10 consecutive samples were removed. The number of trials removed during preprocessing ranged from 11 to 295 per participant, with an average of 132.5 trials (exact number of trials included in the analysis for each participant can be found in Table S2 in the Data S1).

#### 1.7.2 Pupil Size

Missing or invalid data was interpolated using cubic‐spline interpolation if possible, using linear interpolation if cubic‐spline interpolation was not possible (when the segment of missing data was too close to the start or end of a trial), and removed if interpolation was impossible altogether (when data was missing from the start and/or until the end of a trial) or if the period of missing data was longer than 500 ms and thus unlikely to reflect a blink.

Blinks were identified using the *blinkreconstruct* function of EyeLinkParser, which relies on a velocity threshold to identify the onset and offset of a blink (for details see Mathôt and Vilotijević 2022). Next, pupil size was z‐scored for each participant separately across all blocks.

### 1.8 Analysis

#### 1.8.1 Power Estimation

To characterize the frequency power content of the prestimulus interval, we computed the power spectral density (PSD) of the signal, focusing on posterior and parietal channels (O1, O2, Oz, POz, Pz, P3, P4, P7, P8). The epoch used for power estimation spanned from 1 s before to the moment of stimulus onset. The PSD was computed with the compute_psd function, as implemented in MNE (Gramfort et al. 2013), using the default spectral estimation method (multitaper, using DPSS tapers (Slepian 1978)). For further analysis, power was normalized by z‐scoring for each frequency band and participant separately. Finally, the average power over the channels of interest was calculated for the theta (4–8 Hz), alpha (individually determined, as described below) and beta (12–30 Hz) bands.

We also employed a PSD computation to determine individual alpha frequencies (IAF) in the epoched data. However, most subjects did not show a clear peak in the alpha range in the prestimulus epoch. Thus, we followed the same procedure as in Samaha and Postle (2015) of choosing the electrode with maximal power in the 8–13 Hz frequency range to determine the IAF (as the frequency with the maximal power in the chosen electrode). We identified the following subject‐specific electrodes: POz, POz, POz, POz, O1, Pz, Pz, POz, POz, Pz, Pz, Pz, O2, O1, O2, Oz. Finally, we extracted average power in a range of ±1 Hz around the IAF peak to be used in the statistical analysis.

#### 1.8.2 Mediation Analysis

To examine the relative contributions of pupil size and neural activity on detection performance, we used structural equation modeling (SEM) to conduct a mediation analysis using the lavaan (Rosseel 2012) package in R (R Core Team 2021). The aim was to test whether power in the alpha, beta, and theta frequency bands mediates the relationship between pupil size and detection performance. We specified a model that included paths from pupil size to each potential mediator, from each mediator to the dependent variable (in two separate analyses: accuracy and proportion of target‐present responses), and a direct path from pupil size to the dependent variable. Indirect effects were computed as the path coefficients from pupil size to each mediator and from the mediator to the dependent variable, while the direct effect is represented by the path coefficient from pupil size to the dependent variable. The model was fitted separately for each participant. We then conducted both traditional and Bayesian (Dienes 2014) one‐sample t‐tests to evaluate the significance of the parameter (slope) estimates against a null hypothesis of no effect. All models were constructed using the average power and pupil size in the interval spanning from −1.5 s to −0.5 s before stimulus onset.

## 2 Results

### 2.1 Descriptives: Behavioral Performance

The descriptive statistics on behavioral performance measures, including average accuracy (i.e., hits and correct rejections), reaction time (RT), and d‐prime (d') are presented in Table 1. D′ is a measure of perceptual sensitivity, defined as the difference between z‐transformed hit and false alarm rates. Due to some participants having a false‐alarm rate of 0, leading to infinite d' values, we applied a so‐called log‐linear correction (Hautus 1995). This approach involves first adding 0.5 to both the number of hits and false alarms and then adding 1 to the total number of both signal (i.e., stimulus present) trials and noise (i.e., stimulus absent) trials prior to computing d'.

**TABLE 1:** Descriptive Statistics of Behavioral Performance.

Statistic	*M*	SD	Min	Max	*N*
Accuracy	72.07%	6.62	62.08%	84.66%	16
RT (ms)	569.14	97.93	357.49	683.95	16
d'	1.91	0.5	1.15	2.54	16

### 2.2 Descriptives: Pupil Size

**FIGURE 2:**
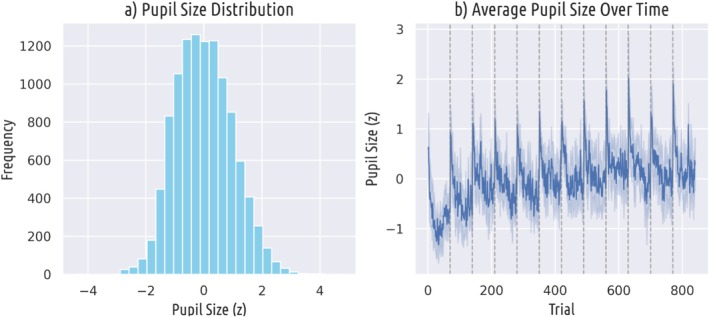
Pupil size descriptives. (A) Distribution of recorded pupil sizes. (B) Average pupil size over time. Dashed vertical lines represent block breaks.

The distribution of z‐scored pupil sizes recorded during the experiment is depicted in Figure 2a. There was a significant positive relationship between average pupil size (across participants) and trial number (*r* = 0.47, *p* < 0.001), indicating that average pupil size increased slightly over the course of the experiment (Figure 2b). Average pupil size was also larger after breaks (indicated by dashed lines in Figure 2b) and then decreased over the course of a block. However, despite variations throughout the block, we included all experimental trials in our analysis to avoid excluding genuine fluctuations in pupil size.

### 2.3 Summary of the Results

**FIGURE 3:**
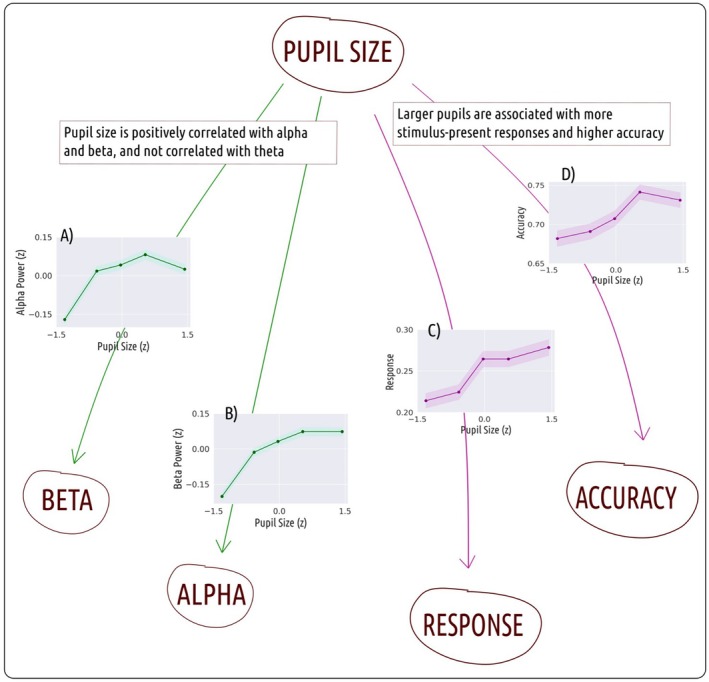
Mediation analysis results. Arrows represent significant effects. Arrows and figures pertaining to the same result are color‐coded. (A) Pupil size is positively correlated with power in the beta frequency band. (B) Pupil size is positively correlated with power in the alpha frequency band. (C) Medium‐to‐large pupil sizes are associated with more stimulus‐present responses. (D) Medium‐to‐large pupil sizes are associated with improved accuracy.

The results of the mediation analysis are graphically summarized in Figure 3. In a nutshell, we found direct effects of pupil size on target‐present responses, accuracy, beta power, and alpha power. We did not find any mediation effects or any direct effects of EEG power on performance (accuracy or target‐present responses). Statistical details are provided below.

In the course of revising this manuscript for publication, we have conducted the full analysis multiple times with slight modifications to the time‐window used and the way power was extracted from the EEG signal. These modifications have also led to slight variations in the pattern of results; the details of different iterations of the analysis can be found in the Data S1. The pupil‐related effects reported here seem particularly robust and have been present at every iteration.

### 2.4 Larger Pupils Are Associated With Improved Accuracy and More Target‐Present Responses

Larger pupils were associated with increased accuracy on single trials (*b* = 0.06, *t*(15) = 2.55, *p* = 0.02; Figure 3c). Larger pupils were also associated with an increased proportion of target‐present responses (*b* = 0.11, *t*(15) = 3.87, *p* = 0.002; Figure 3b).

These results were corroborated by the Bayesian analysis, showing moderate to strong evidence for the alternative hypotheses (i.e., path coefficient differs from zero). The direct pathway from pupil size to accuracy showed a Bayes Factor of 2.84, while the direct pathway from pupil size to response showed a Bayes Factor of 27.01.

### 2.5 Pupil Size Is Positively Correlated With Alpha and Beta Power, and Not Correlated With Theta Power

Larger pupils were associated with increased power in the beta and alpha bands (Figure 3a; 3b) but there was no relationship with power in the theta band. The direct effects from pupil size to beta power (*b* = 0.09, *t*(15) = 3.67, *p* = 0.002) and pupil size to alpha power (*b* = 0.06, *t*(15) = 2.45, *p* = 0.027) were significantly different from zero, but the direct effect from pupil size to theta power (*b* = 0.01, *t*(15) = 0.49, *p* = 0.63) was not.

The Bayesian analysis provided strong evidence for the alternative hypothesis (i.e., path coefficient differs from zero) for the relationship between pupil size and beta, and moderate evidence for the alternative hypothesis for the relationship between pupil size and alpha. The path coefficient from pupil size to beta power showed a Bayes Factor of 18.91, and the path coefficient from pupil size to alpha power showed a Bayes Factor of 2.46. For the relationship between pupil size and theta power, on the other hand, the Bayes Factor in favor of the alternative hypothesis was 0.28, indicating weak support.

### 2.6 The Effects of Pupil Size on Performance Are Not Mediated by Power in the EEG

The indirect effects in our mediation model with accuracy as a dependent variable did not significantly differ from zero. This was indicated both by traditional one‐sample t‐tests and Bayesian t‐tests. Specifically, the Bayesian analysis provided moderate evidence for the null hypothesis for all indirect pathways. The path coefficient from pupil size to accuracy via beta power showed a Bayes Factor of 3.88 in favor of the null, the path through alpha power showed a Bayes Factor of 1.21 in favor of the null, and the path through theta power showed a Bayes Factor of 3.33 in favor of the null, all suggesting the absence of significant indirect effects.

These results indicate that the relationship between pupil size and accuracy was not mediated by power in any of the measured frequency bands.

Similarly, the indirect effects in our mediation model with response as the dependent variable did not significantly differ from zero, as indicated by both traditional one‐sample t‐tests and Bayesian t‐tests. Bayesian analyses provided moderate evidence for the null hypothesis for all indirect pathways. The path coefficient from pupil size to response via beta power showed a Bayes Factor of 1.53 in favor of the null, the path through alpha power showed a Bayes Factor of 2.83 in favor of the null, and the path through theta power showed a Bayes Factor of 3.6 in favor of the null, all suggesting the absence of significant indirect effects. These results indicate that the relationship between pupil size and response was not mediated by power in any of the measured frequency bands.

## 3 Discussion

Here, we have investigated the effects of pupil size and neural activity on visual detection performance. We observed that increased pupil size is associated with improved detection accuracy and more target‐present responses. The effect of pupil size on performance was not mediated by power in any of the examined EEG bands. However, we have shown a positive correlation between pupil size and power in the beta and alpha bands, consistent with previous research (Mathôt et al. 2023; Montefusco‐Siegmund et al. 2022; Pilipenko and Samaha 2024; Podvalny et al. 2021; Waschke et al. 2019). Our findings suggest a complex interplay between pupil size, arousal, and visual detection, revealing a network of relationships that are less straightforward than previously thought.

Our mediation analysis considered two single‐trial dependent measures: accuracy (whether the response was correct) and target‐present response (whether the target was reported as present regardless of whether it actually was). Due to the nature of our task, where participants only give a response if they detect a stimulus, the single‐trial responses roughly map onto the signal‐detection measure of criterion, such that a high number of target‐present responses indicates a lower criterion. Furthermore, since false alarm rates in our sample were on average low (< 5%), overall accuracy roughly maps onto sensitivity (d') such that higher accuracy indicates higher sensitivity. Based on this, for the remainder of this section, we will discuss our results in terms of sensitivity and criterion. While our measures do not directly reflect these constructs, they give a good indication of potential relationships that may be further investigated in future work. We have visualized the pattern of our mediation analysis results in terms of sensitivity and criterion in Figure S1 of the Data S1.

Contrary to expectations based on previous research (Ergenoglu et al. 2004; Montefusco‐Siegmund et al. 2022; Pilipenko and Samaha 2024; Samaha et al. 2017), we found no significant relationship between alpha power and detection performance (neither sensitivity nor criterion). Some authors have suggested that prestimulus alpha phase is more relevant in determining stimulus detectability than alpha power is (Busch et al. 2009; Mathewson et al. 2009; van Dijk et al. 2008), which may explain our findings. Furthermore, Pilipenko and Samaha (2024) show dissociable effects of alpha power and pupil size on detection, whereby variations in alpha power affect the criterion (Iemi et al. 2017; Samaha et al. 2017; Samaha et al. 2020) while variations in pupil size affect sensitivity. As mentioned in the methods section, the false alarm rates of our participants were generally low (< 5%) and there was not much variation between participants (see Data S1 for details); it may be that our task did not induce measurable criterion shifts, which could explain the null finding on alpha power. Theta and beta power changes, on the other hand, are not generally linked to detection performance. They were included here for exploratory analysis, so their lack of association with task performance is less surprising.

The key finding of our study is that larger pupils benefit detection partly independently of the changes observed in the EEG power spectrum. Pupil‐size related effects on visual detection are often overlooked, and thus, for the remainder of this section, we will focus on discussing these results. The improvement in visual sensitivity that results from increased pupil size can be explained in two ways (and is likely to be a combination of both mechanisms). Firstly, larger pupils allow more light into the eye, thus decreasing the signal‐to‐noise ratio and improving sensitivity (Mathôt 2020), which we call the optical effect. Secondly, larger pupils reflect variations in arousal (or cortical excitability), which affects visual sensitivity by changing the way the stimulus is processed, which we call the arousal effect. Dissociating these effects is not trivial and is further complicated by the lack of consensus on what ‘arousal’ is and which measures reflect it in which ways (Ross and Van Bockstaele 2021; Eysenck 1976; Robbins 1997).

Another complicating factor in dissociating these effects is that the relationship between pupil size and arousal is more nuanced than previously understood. Traditionally viewed as a direct marker of arousal due to its association with locus coeruleus (LC) activity (Joshi et al. 2016; Murphy et al. 2014), recent findings suggest this connection is more variable (Megemont et al. 2022). Research done in mice by Megemont et al. (2022) reveals that only large and infrequent pupil dilations correspond directly with spiking activity in the LC, challenging the notion of pupil size as a consistent readout of LC activity. Additionally, variability in this relationship might be influenced by behavioral factors, highlighting inconsistencies in how pupil dilation reflects arousal states. Furthermore, another study examining arousal‐related neural activity and pupil size in mice found that dynamic changes in pupil size, rather than absolute size, align more closely with neural activity changes associated with different arousal states in the dorsal lateral geniculate nucleus (dLGN). More specifically, tonic spiking (associated with wakefulness) occurred more during pupil dilation, while burst spiking (associated with low‐arousal states) occurred more during pupil contraction (Crombie et al. 2024). Interestingly, these rhythms associated with arousal‐state regulation have also been associated with the generation of alpha‐like rhythms in mice (Nestvogel and McCormick 2022). These findings underscore the complexity in linking pupil size directly to arousal‐related neural activity (for more discussion on the neural and arousal‐related correlates of pupil size, see Grujic et al. 2024).

Somewhat similarly, while changes in the EEG frequency spectrum have been used as markers of cortical arousal (Danos et al. 2001; Foucher et al. 2004) or cortical excitability (Pilipenko and Samaha 2024), different studies use different terms (e.g., arousal, excitability, activation) and define different EEG correlates. Some treating arousal and cortical excitability as interchangeable terms, while others do not, is one reflection of the lack of a clear definition of arousal as a unitary concept (Eysenck 1976). However, insofar as amplitude differences in the lower frequencies can be considered to reflect arousal variation (Foucher et al. 2004), our findings demonstrate that these differences do not explain the relationship between pupil size and detection performance. This supports the assumption that at least a part of the large‐pupil advantage observed in near‐threshold detection is due to an optical effect driven by pupil dilation itself.

Additional evidence for the optical effect comes from behavioral experiments measuring pupil size in detection and discrimination tasks, as well as pupil‐size measurements obtained in experiments on auditory near‐threshold detection. As previously mentioned, in a study involving both detection and discrimination, average pupil size was found to be larger in the detection condition, despite no obvious differences in task difficulty (Mathôt and Ivanov 2019). The lack of a difference in difficulty implies that there should be no systematic differences in applied mental effort or arousal. While it is theoretically possible that visual detection tasks are inherently more arousing than discrimination tasks, in a standard lab‐setting, this seems unlikely. Furthermore, in a recent experiment on perceptual awareness of near‐threshold tones, pupil dilation exhibited an inverted‐U shaped relationship with detection rate (i.e., hit rate), whereby detection was optimal at intermediate pupil sizes (Doll et al. 2024). This pattern aligns with the Yerkes‐Dodson law, which posits that moderate arousal levels optimize performance (Yerkes and Dodson 1908). Importantly, we and others (Eberhardt et al. 2022; Mathôt and Ivanov 2019) have observed a relationship between pupil size and hit rate and/or accuracy (not sensitivity) that has a linear component, whereby larger pupils are associated with better detection performance.

Specifically, referring to Figure 3d, in our data, the relationship between pupil size and accuracy resembles a combination of an inverted U shape, which may be driven by arousal, and a linear component, which we believe is driven by the optical effect resulting from an increase in pupil size.

Finally, we have shown positive correlations between pupil size and power in both the alpha and beta bands, replicating findings from previous research (Ceh et al., 2020; Hong et al. 2014; Mathôt et al. 2023; Montefusco‐Siegmund et al. 2022; Pilipenko and Samaha 2024, Waschke et al. 2019). Alpha power has been more frequently studied, likely because both alpha power and pupil size are linked to similar processes such as visual perception, attention, and arousal. However, our results show a stronger correlation between pupil size and beta power, in line with observations by Mathôt et al. (2023) and Waschke et al. (2019). Considering the known association of beta power with motor activity (Engel and Fries 2010; Jenkinson and Brown 2011), one possibility is that this relationship is a reflection of oculomotor control of the iris dilator muscle. Higher beta power has been associated with more tonic contractions of the muscles (Jenkinson and Brown 2011), which in the iris dilator muscle manifests as a dilation of the pupil. While the exact implications of this relationship remain unclear, its replicability offers a promising direction for further research into the interplay between pupil size and EEG activity patterns.

In conclusion, here we have shown that larger prestimulus pupil size is associated with enhanced sensitivity in near‐threshold detection. Importantly, this relationship is not mediated by power in the EEG, suggesting an independent influence of pupil size on visual detection. Additionally, we observed a positive correlation between pupil size and power in the alpha and beta bands. Our results highlight pupil size changes as a crucial yet still poorly understood factor in visual processing.

## Author Contributions

**Veera Ruuskanen:** conceptualization, data curation, formal analysis, investigation, methodology, software, validation, visualization, writing – original draft, writing – review and editing. **C. Nico Boehler:** funding acquisition, methodology, project administration, resources, supervision, validation, writing – review and editing. **Sebastiaan Mathôt:** conceptualization, data curation, formal analysis, funding acquisition, methodology, project administration, resources, supervision, validation, writing – review and editing.

## Conflicts of Interest

The authors declare no conflicts of interest.

## Supporting information


Data S1.

## Data Availability

The data and analysis code is available on the OSF and Github: https://osf.io/9p563/.
