# Supplementary material for: The Interplay of Spontaneous Pupil‐Size Fluctuations and EEG Power in Near‐Threshold Detection
Source: Psychophysiology. 2025 Mar 16;62(3):e70035. doi: 10.1111/psyp.70035 (PMC11911296; doi:10.1111/psyp.70035)
Supplement: Supplementary file 1 — Data S1. [file PSYP-62-e70035-s001.pdf]

Supplementary figures and analyses associated with Ruuskanen et al., 2024, The interplay of spontaneous pupil-size fluctuations and EEG power in near-threshold detection

Cross-correlation analysis pupil size, alpha and beta power.

Since pupil size changes may lag behind in time from associated neural events, we conducted an additional time-lagged correlation analysis on the relationship between pupil size and power in the alpha and beta bands. The same trials were included as in the main analysis, and alpha and beta power were lagged by one trial. Thus, the results pertain to the correlation of pupil size on trial *t* and alpha/beta power on trial *t-1*.

The resulting sample of correlations (Table 1) was Fisher transformed and tested with a one-sample t-test. The alternative hypothesis was that the population correlation coefficient is different from 0. For the correlation between pupil size and beta power the test was significant ($t(15) = 2.6, p = 0.02$) but for the correlation between pupil size and alpha power it was not ($t(15) = 1.2, p = 0.25$).

Table 1

Time-lagged correlation between pupil size and power in the alpha and beta bands

Subject	beta_pupil	beta_pupil_p	alpha_pupil	alpha_pupil_p
1	0.159	0.000	0.063	0.079
2	0.051	0.159	-0.013	0.708
3	0.100	0.005	-0.053	0.139
4	0.124	0.002	0.063	0.127
5	-0.167	0	-0.006	0.858
6	0.275	0	0.212	0

7	0.130	0.000	0.070	0.060
8	0.180	0	0.064	0.082
9	-0.140	0.000	-0.222	0
10	0.085	0.050	0.140	0.001
12	0.124	0.000	0.059	0.112
13	0.218	0	0.027	0.485
15	0.123	0.002	0.250	0
16	-0.025	0.558	-0.035	0.407
17	0.077	0.031	-0.032	0.364
19	-0.051	0.152	-0.0482	0.179

Additional information about data quality and performance for each subject.

Table 2 includes the number of trials included in the analysis for each subject (out of a possible total of 840) and the False Alarm rate for each subject, computed as the proportion of target-absent trials where a participant still reported seeing a target.

Table 2

Subject specifics: number of trials included in analysis and false-alarm rate

Subject	N trials	FA rate
1	808	0
2	782	0.012
3	829	0.005
4	769	0.014
5	726	0.019
6	675	0.034

7	546	0.119
8	772	0.043
9	727	0.002
10	672	0.033
12	701	0.043
13	655	0
15	589	0.029
16	545	0.236
17	785	0.007
19	738	0.029

Details of different iterations of the analysis.

As mentioned in the manuscript on page 18 (section Summary of the results) the analysis has been carried out multiple times with slight modifications throughout the review process. Here, we report the details of the analyses and their results (Table 3).

The channels of interest and the basic preprocessing (as reported in the manuscript) were the same for each iteration of the analysis. The specification of the mediation analysis as well as how the significance of effects was tested also stayed the same.The differences lie in the way power was extracted from the EEG (wavelet analysis vs. power spectral density computation) and the exact time-window used.

Analysis A. In the first iteration of the analysis we computed the power spectrum for single trials across a frequency range of 4 Hz to 30 Hz with a fast-fourier transform using Morlet wavelets, as implemented in MNE (function tfr_morlet) (Gramfort et al., 2013). The epoch used

in the time-frequency analysis spanned from 2.5 s before to 0.5 s after stimulus onset. For further analysis, power was normalised by z-scoring for each frequency band and participant separately, and the epoch was cropped (to avoid filtering edge artefacts) to span 1 s before stimulus presentation.

Analysis B. In analysis B we again used Morlet wavelets to characterize the power content of single trials. However, this time the epoch spanned from 2.5 s before to the moment of stimulus onset, to avoid including stimulus-evoked activity. Power was normalised by z-scoring for each frequency band and participant separately, across trials, channels and timepoints. Then, the epoch was cropped (to avoid edge artefacts) to span from -1.5 seconds to -0.5 seconds relative to stimulus onset. In analysis B we also used the individual alpha frequency, extracted as defined in the manuscript on page 14 (section Power estimation).

Analysis C. For the final iteration, we dropped the time-frequency approach and instead simply computed the power spectral density as described in the manuscript on page 14 (section Power estimation). This way we did not need to crop the signal to avoid edge artefacts, and were able to focus our analysis on the activity in the 1 second preceding stimulus presentation.

Table 3

Average slope estimates and associated p-values for each effect of interest at each iteration of the analysis. Cells with a green background are significant effects.

effect	slope_A	p_A	slope_B	p_B	slope_C	p_C
pupil_theta	-0.006	0.750	-0.016	0.401	0.015	0.631
pupil_alpha	**0.055**	**0.030**	**0.044**	**0.063**	**0.063**	**0.027**

pupil_beta	**0.056**	**0.001**	**0.045**	**0.006**	**0.096**	**0.002**
pupil_acc	**0.023**	**0.012**	**0.060**	**0.020**	**0.066**	**0.022**
pupil_response	**0.032**	**0.001**	**0.029**	**0.003**	**0.110**	**0.002**
theta_acc	**-0.032**	**0.003**	**-0.112**	**0.011**	**-0.056**	**0.167**
alpha_acc	0.003	0.790	0.035	0.219	0.025	0.564
beta_acc	-0.002	0.805	-0.010	0.786	-0.023	0.265
theta_response	**-0.024**	**0.010**	**-0.028**	**0.001**	**-0.056**	**0.185**
alpha_response	0.006	0.552	0.012	0.246	0.015	0.714
beta_response	**-0.034**	**0.032**	**-0.027**	**0.099**	**-0.045**	**0.073**
pupil_theta_acc	-0.000	0.959	**0.004**	**0.039**	**-0.003**	**0.552**
pupil_alpha_acc	-0.000	0.420	0.003	0.121	0.007	0.110
pupil_beta_acc	0.000	0.454	-0.001	0.599	-0.000	0.897
pupil_theta_response	-0.000	0.709	0.000	0.574	-0.002	0.671
pupil_alpha_response	0.001	0.158	0.002	0.082	0.006	0.401
pupil_beta_response	**-0.001**	**0.047**	**-0.002**	**0.030**	**-0.006**	**0.153**

Mediation analysis results with figures depicting relationships between measured variables and criterion and sensitivity (d'). To compute criterion and d', trials have been binned according to either pupil size or theta power (5 bins).

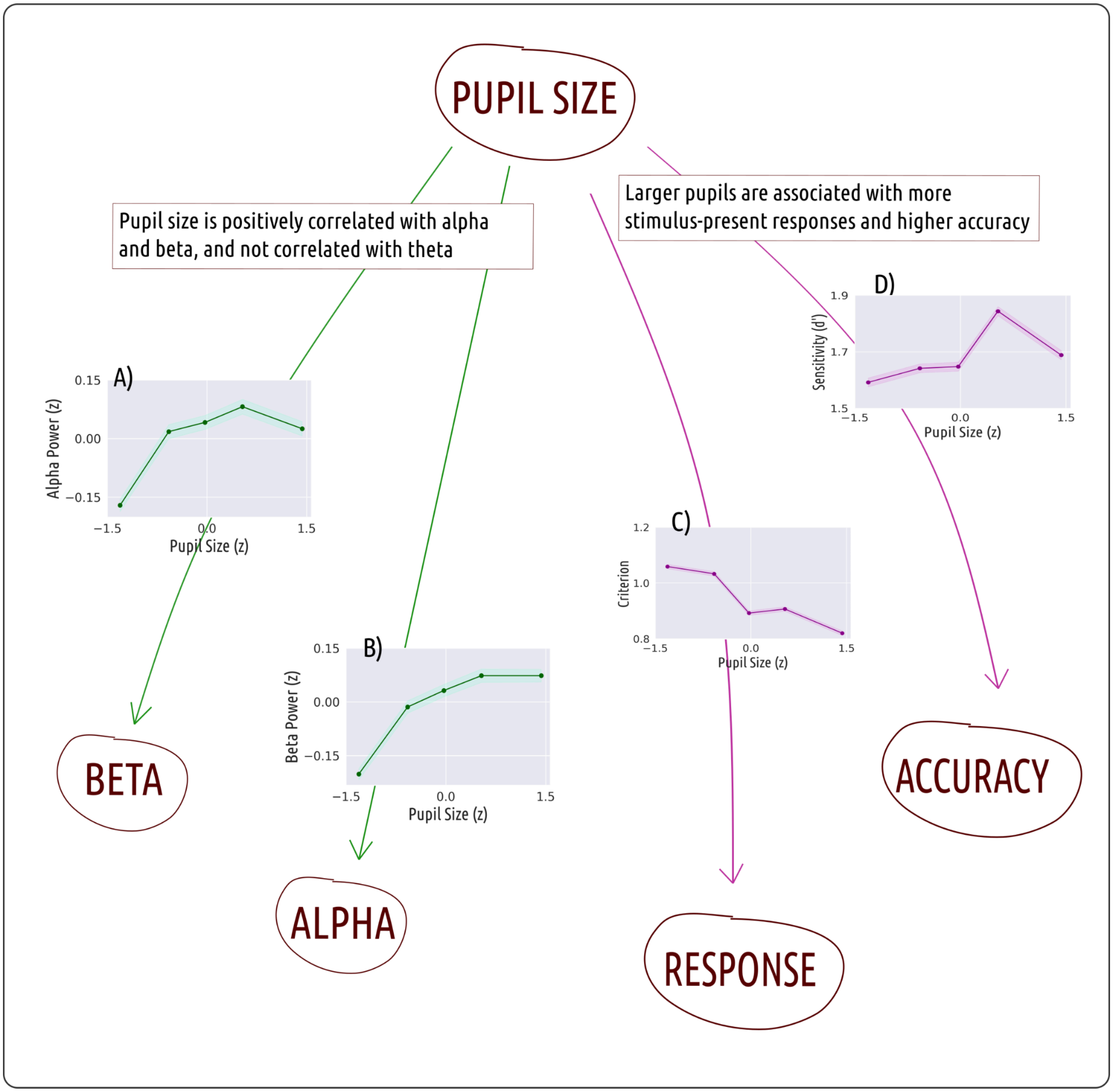

Figure 1: Mediation analysis results visualised in a signal-detection theory framework. Arrows represent significant effects; solid lines are direct effects. Arrows and figures pertaining to the same result are colour-coded. A) Pupil size is positively correlated with power in the beta frequency band. B) Pupil size is

positively correlated with power in the alpha frequency band. C) Medium-to-large pupil sizes are associated with a more liberal criterion. D) Medium-to-large pupil sizes are associated with improved sensitivity.

Depiction of the time-frequency content of the clipped pre-stimulus interval.

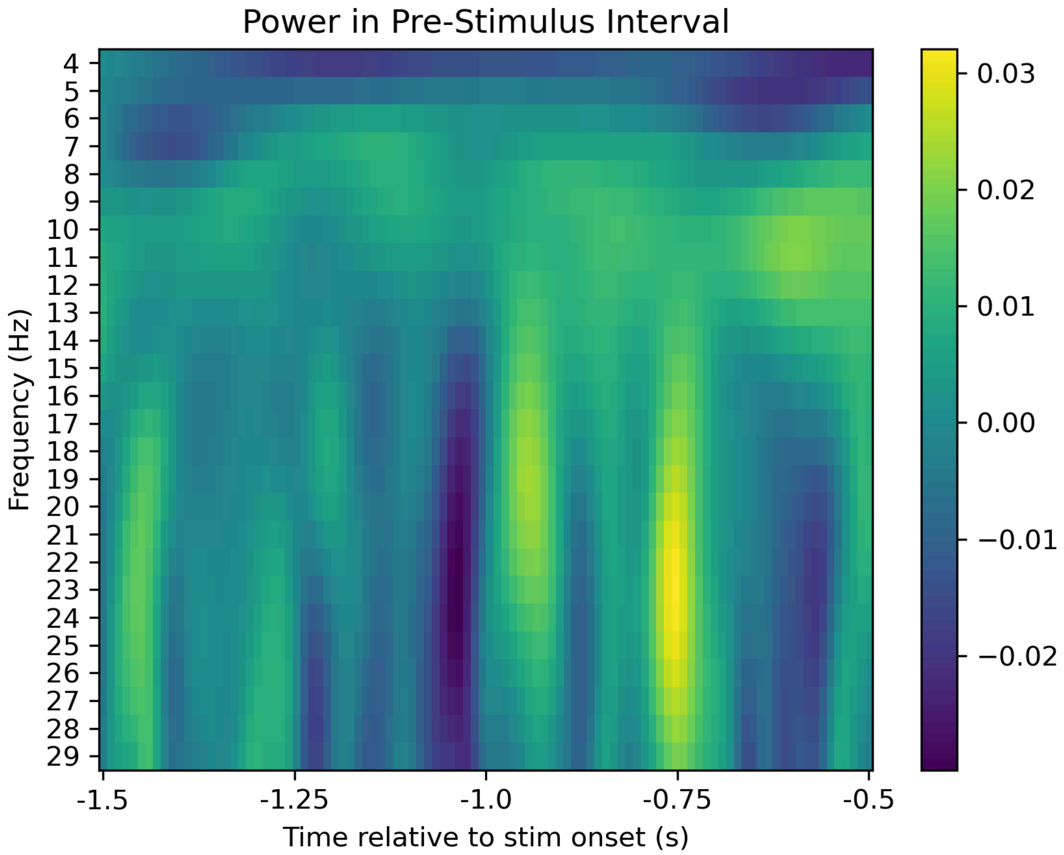

Figure 2: Time-frequency spectrum depicting power variation in the pre-stimulus interval. This figure is based on the TFR analysis described above in Analysis B, and thus, the time-frame is slightly different to the final analysis (Analysis C) which is based on the 1 second preceding the presentation of the stimulus. We chose to use the cropped timeframe so as to not depict edge artifacts or stimulus-evoked activity in this figure. Note that higher frequencies are at the bottom of the figure and lower frequencies at the top.

Power spectral density in the 1-30 Hz range in channels of interest

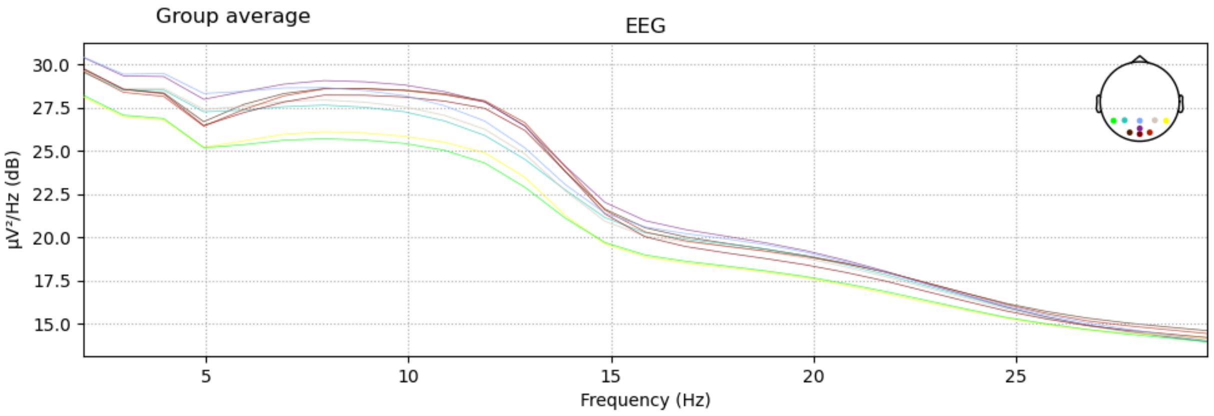

Figure 3: Average power spectral density of all subjects in the pre-stimulus epoch. Channels included are O1, O2, Oz, POz, Pz, P3, P4, P7, P8.

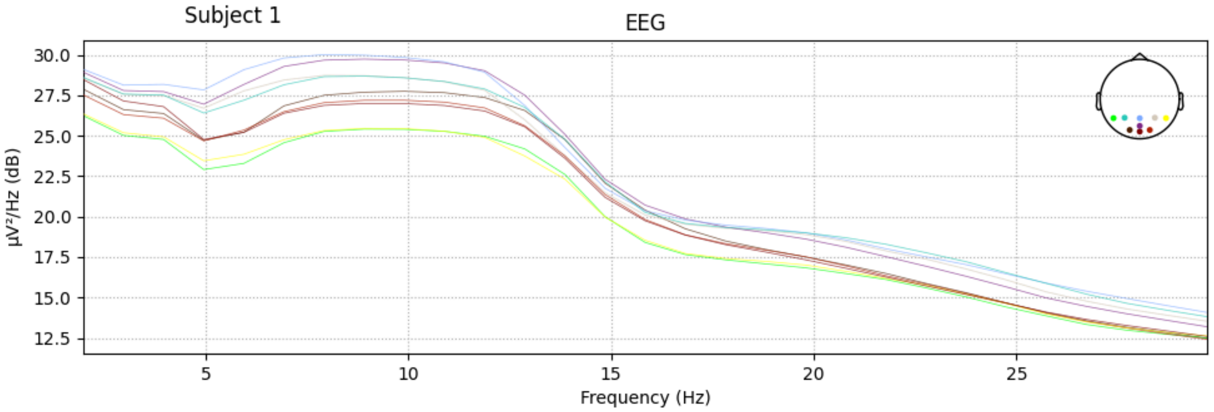

Figure 4: Average power spectral density of subject 1 in the pre-stimulus epoch.

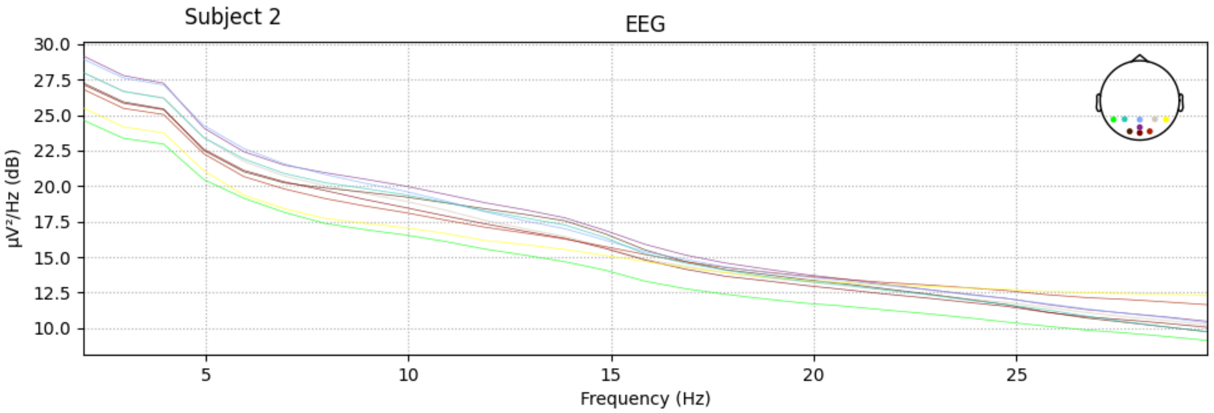

Figure 5: Average power spectral density of subject 2 in the pre-stimulus epoch.

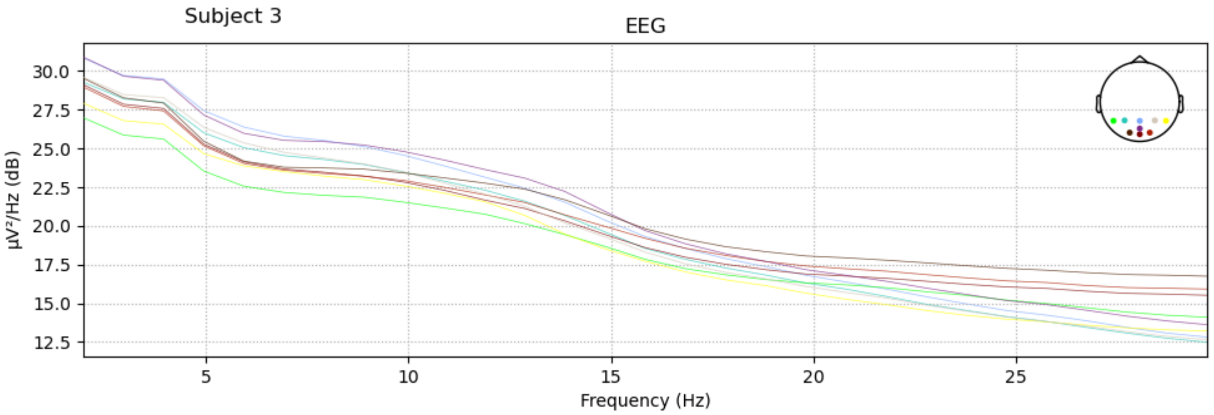

Figure 6: Average power spectral density of subject 3 in the pre-stimulus epoch.

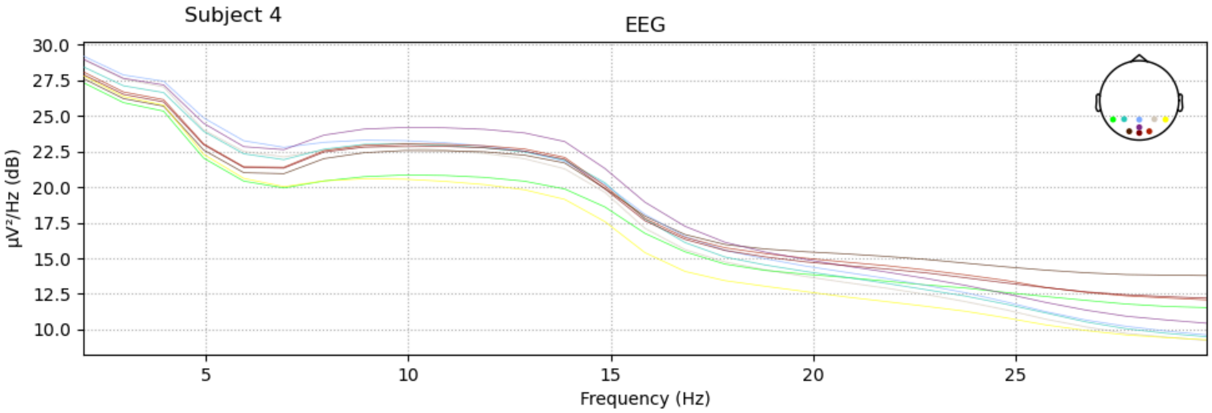

Figure 7: Average power spectral density of subject 4 in the pre-stimulus epoch.

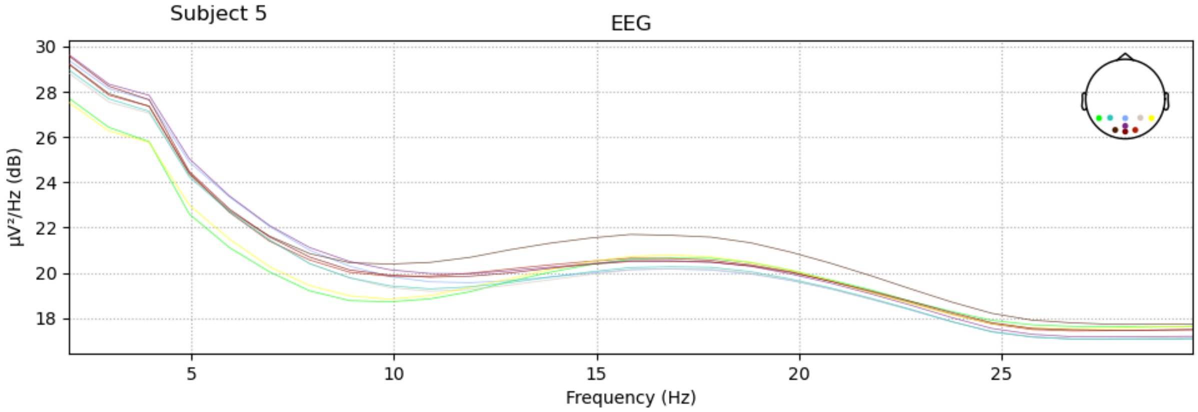

Figure 8: Average power spectral density of subject 5 in the pre-stimulus epoch.

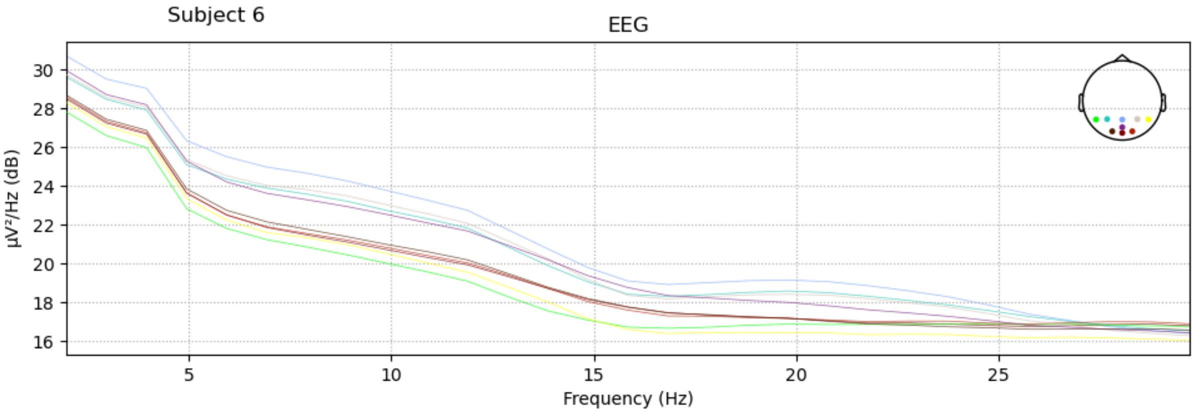

Figure 9: Average power spectral density of subject 6 in the pre-stimulus epoch.

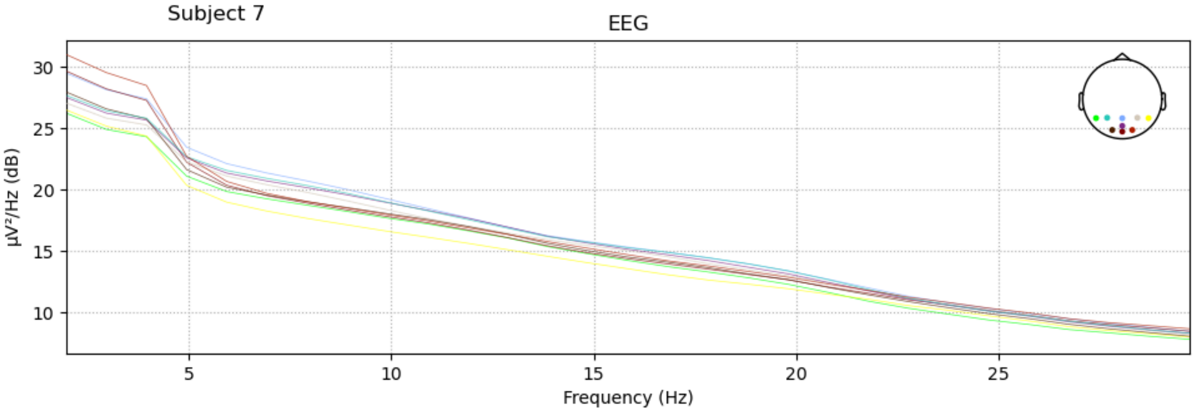

Figure 10: Average power spectral density of subject 7 in the pre-stimulus epoch.

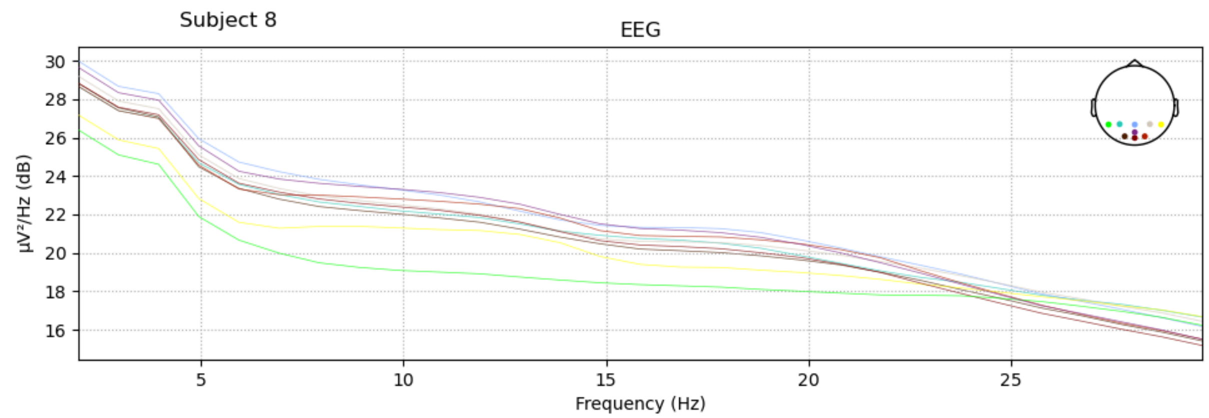

Figure 11: Average power spectral density of subject 8 in the pre-stimulus epoch.

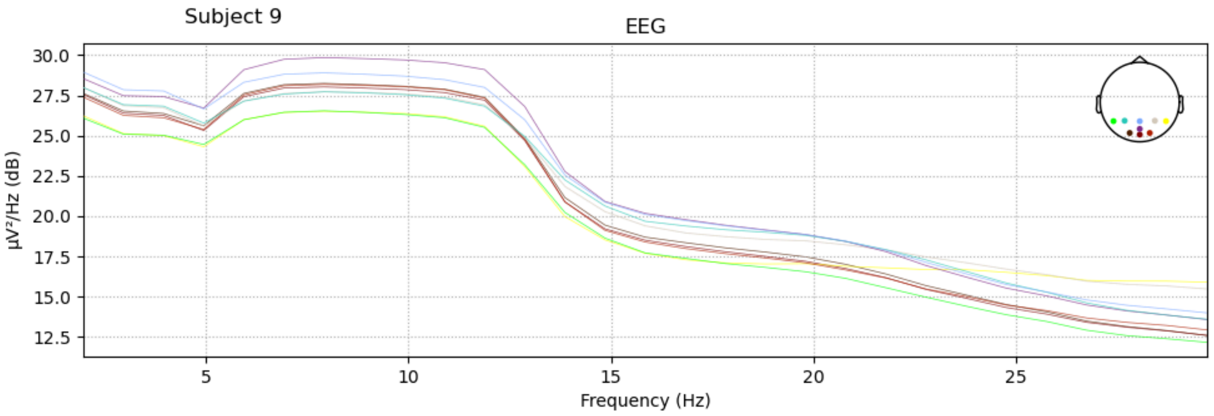

Figure 12: Average power spectral density of subject 9 in the pre-stimulus epoch.

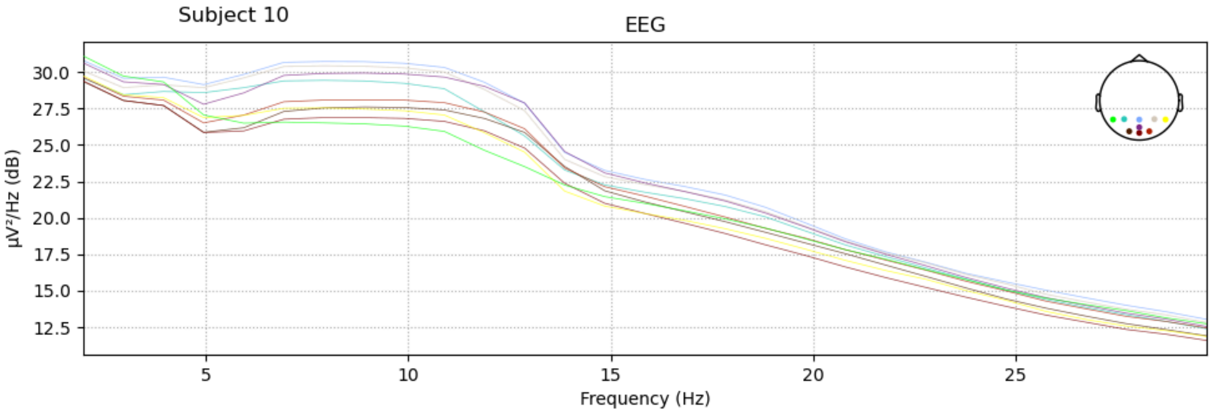

Figure 13: Average power spectral density of subject 10 in the pre-stimulus epoch.

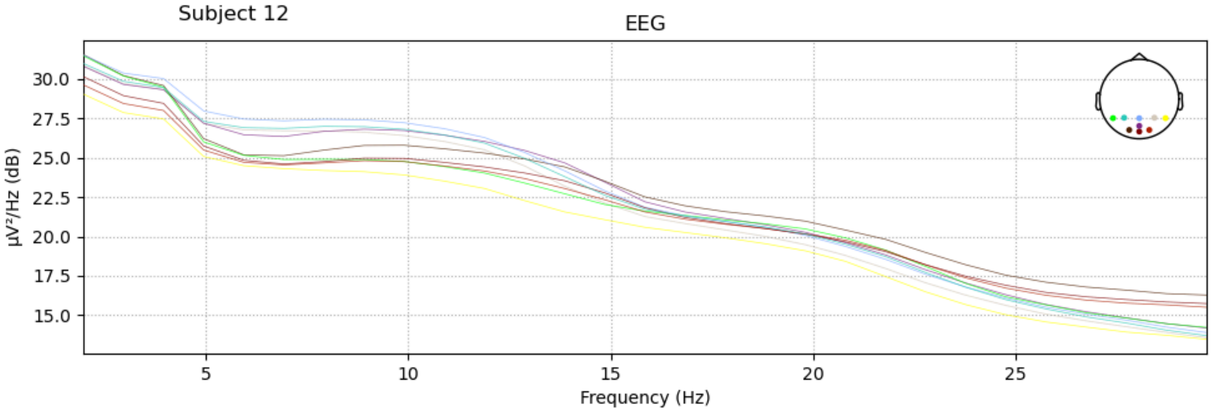

Figure 14: Average power spectral density of subject 12 in the pre-stimulus epoch.

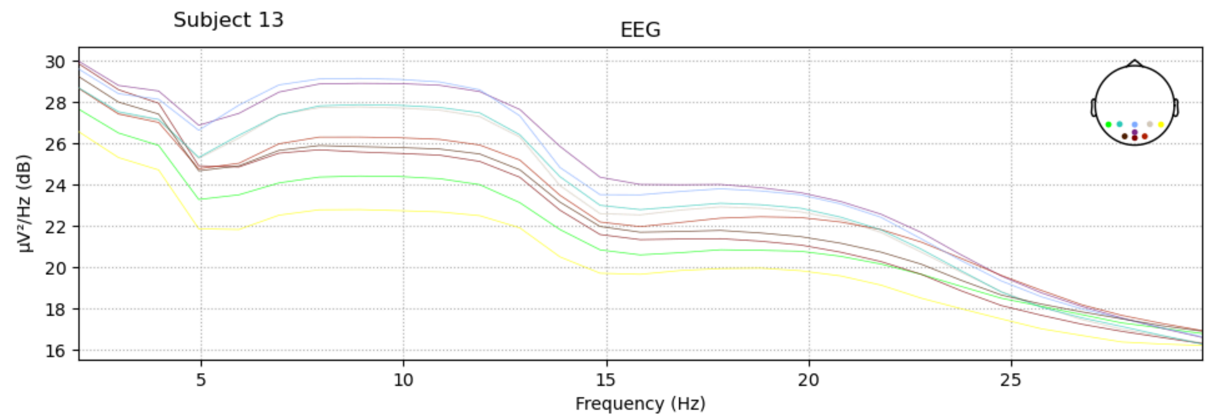

Figure 15: Average power spectral density of subject 13 in the pre-stimulus epoch.

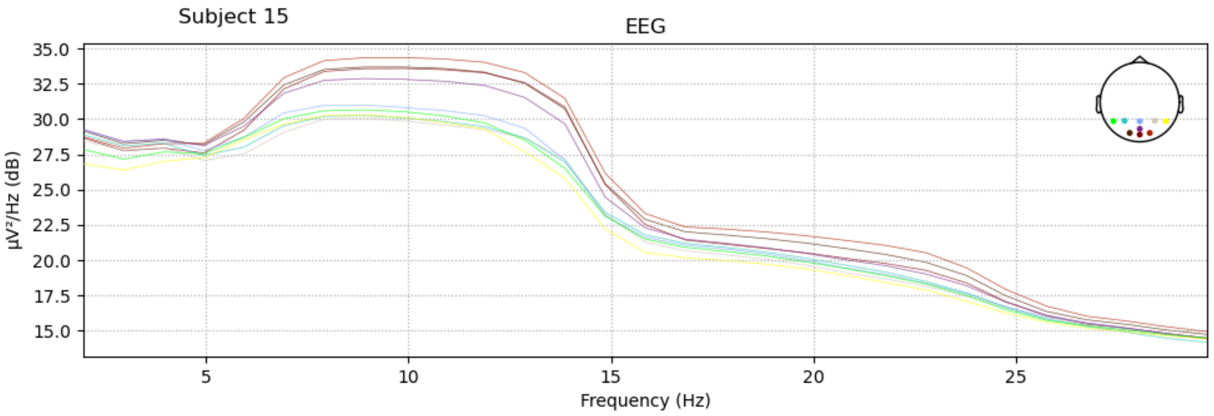

Figure 16: Average power spectral density of subject 15 in the pre-stimulus epoch.

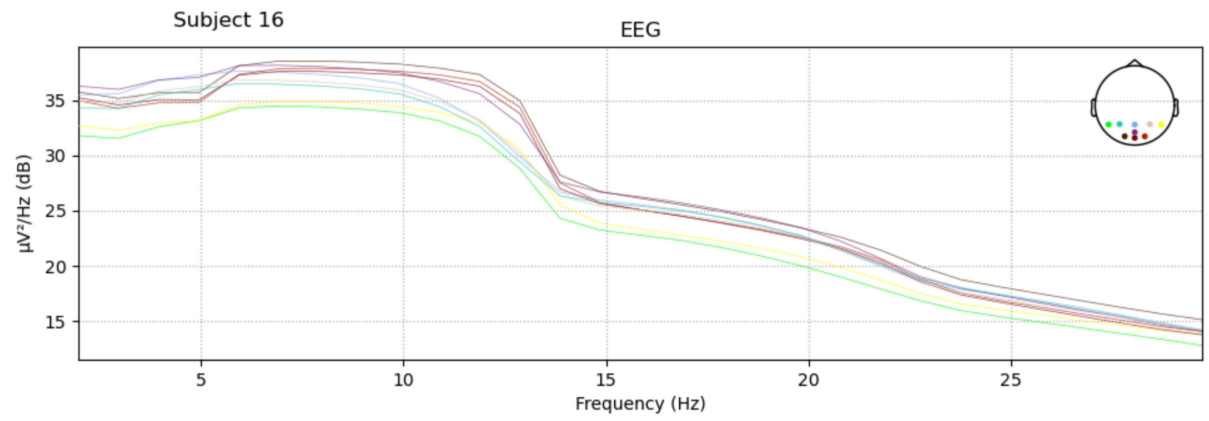

Figure 17: Average power spectral density of subject 16 in the pre-stimulus epoch.

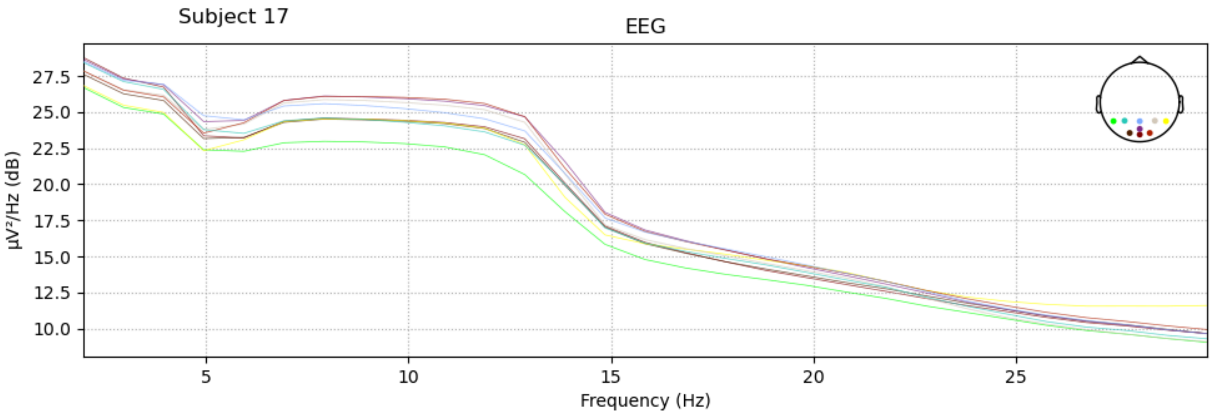

Figure 18: Average power spectral density of subject 17 in the pre-stimulus epoch.

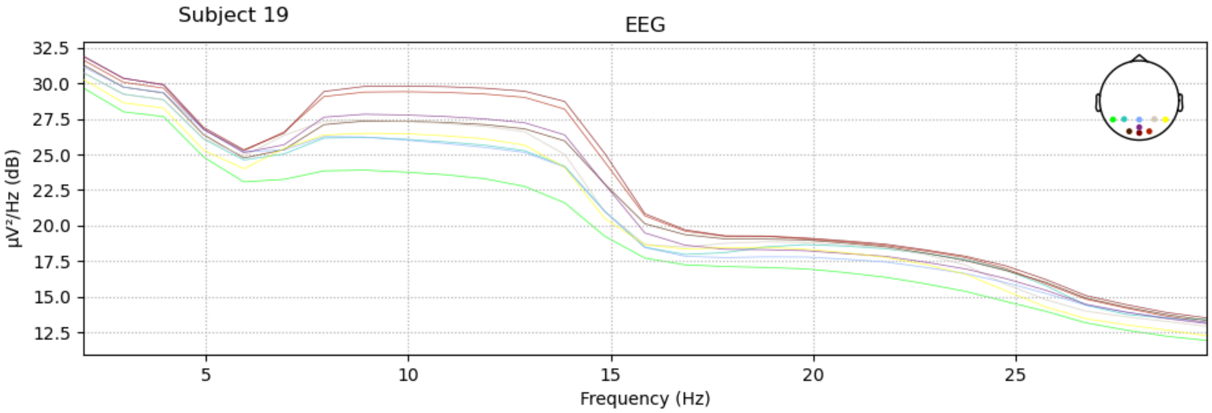

Figure 19: Average power spectral density of subject 19 in the pre-stimulus epoch.

Topographic maps of PSD in all channels

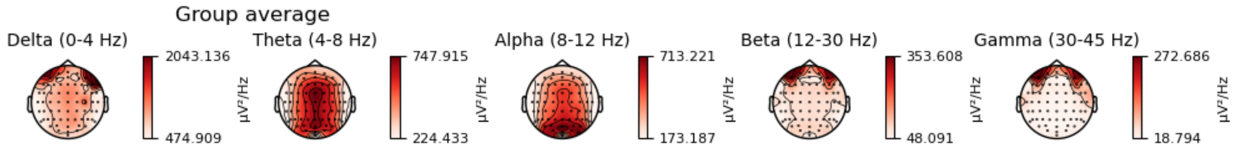

Figure 20: Topographic map of the frequency content of all channels averaged across all subjects.

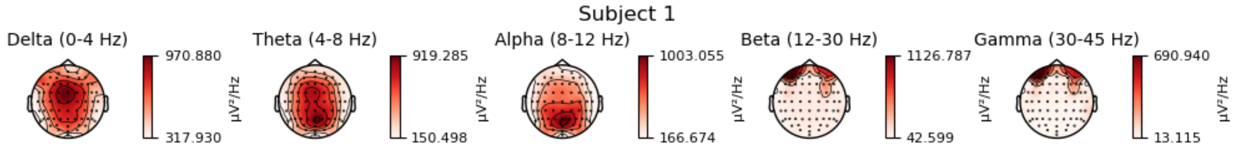

Figure 21: Topographic map of the frequency content of all channels for subject 1.

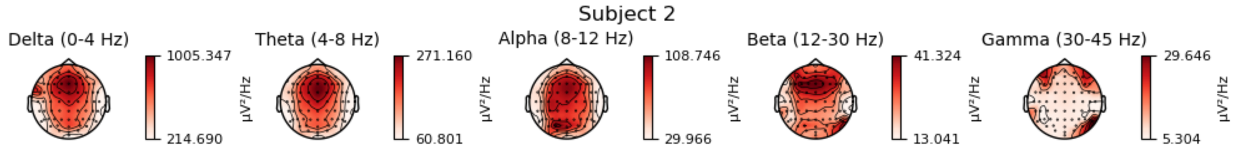

Figure 22: Topographic map of the frequency content of all channels for subject 2.

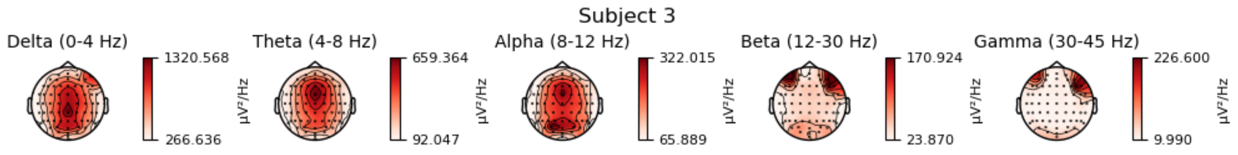

Figure 23: Topographic map of the frequency content of all channels for subject 3.

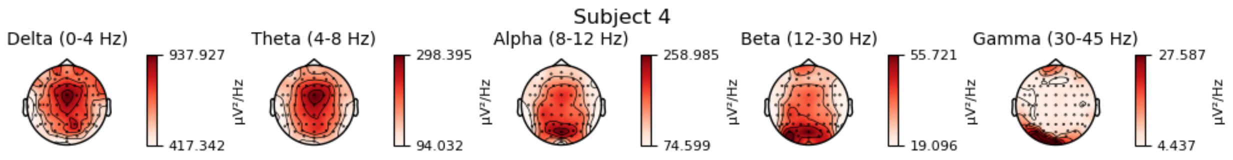

Figure 24: Topographic map of the frequency content of all channels for subject 4.

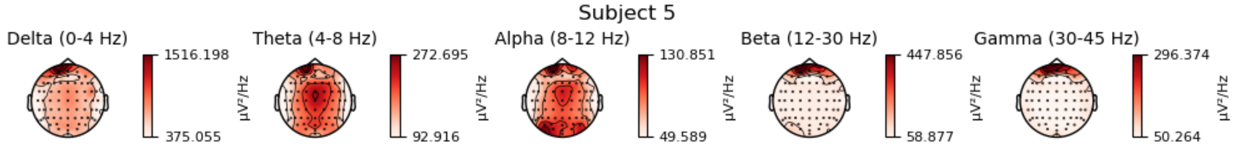

Figure 25: Topographic map of the frequency content of all channels for subject 5.

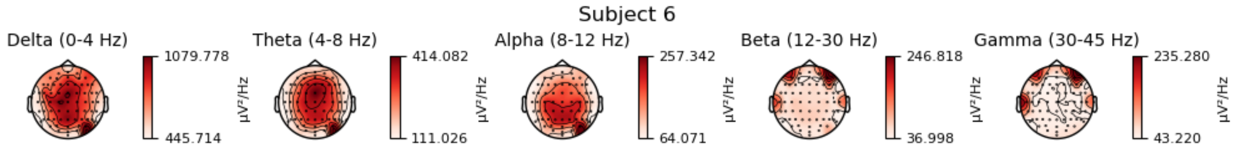

*Figure 26:*Topographic map of the frequency content of all channels for subject 6.

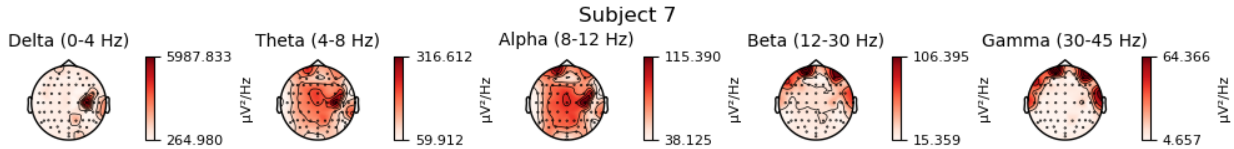

Figure 27: Topographic map of the frequency content of all channels for subject 7.

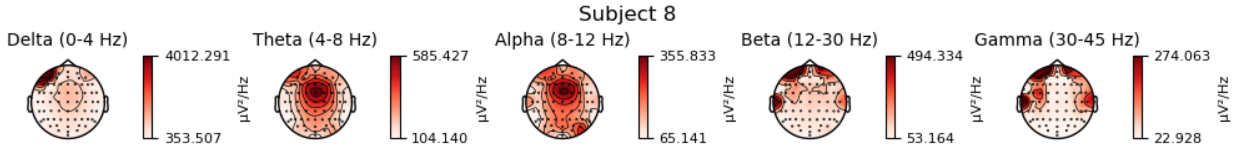

Figure 28: Topographic map of the frequency content of all channels for subject 8.

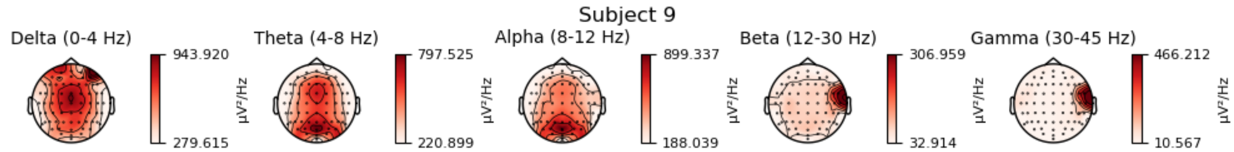

Figure 29: Topographic map of the frequency content of all channels for subject 9.

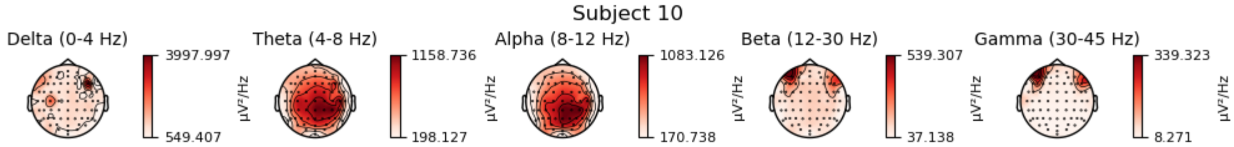

Figure 30: Topographic map of the frequency content of all channels for subject 10.

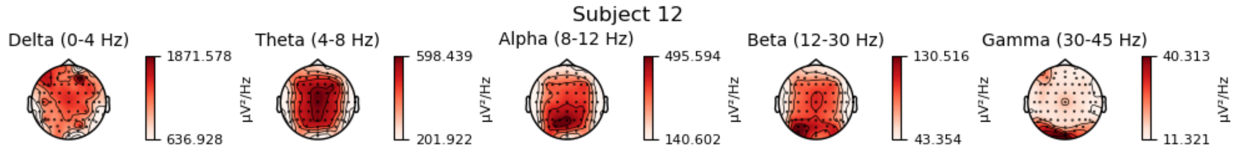

Figure 31: Topographic map of the frequency content of all channels for subject 12.

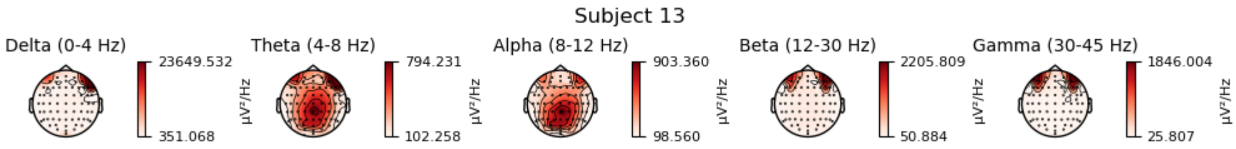

Figure 32: Topographic map of the frequency content of all channels for subject 13.

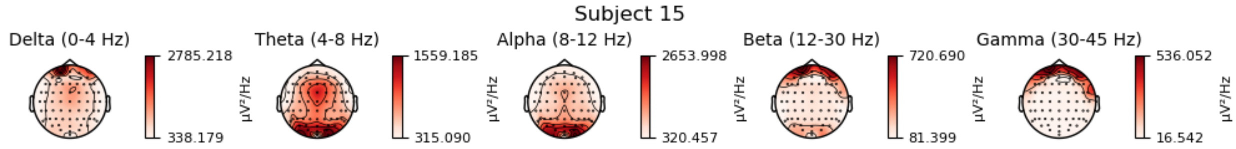

Figure 33: Topographic map of the frequency content of all channels for subject 15.

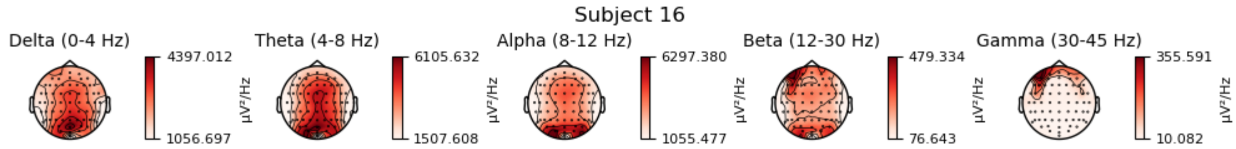

Figure 34: Topographic map of the frequency content of all channels for subject 16.

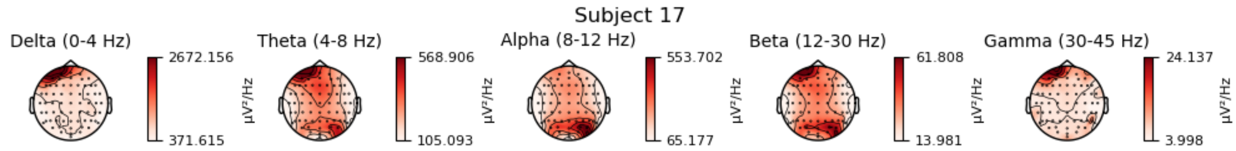

Figure 35: Topographic map of the frequency content of all channels for subject 17.

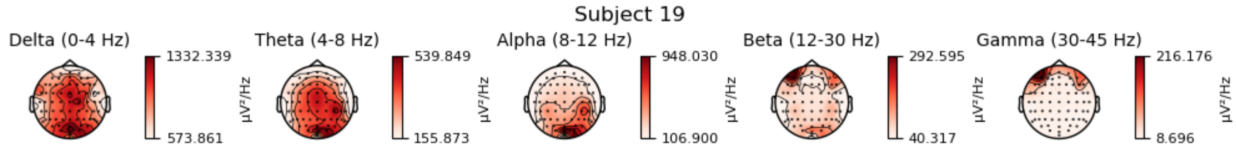

Figure 36: Topographic map of the frequency content of all channels for subject 19.
